# An exploration of the value of NLR, PLR, LMR, and WBC × CRP for the diagnosis and treatment of influenza B in adults

**DOI:** 10.1097/MD.0000000000037046

**Published:** 2023-02-02

**Authors:** Juan-Fei Qi, Mei-Li Guo, Li Lin, Shui Fu, Liu-Ling Chen

**Affiliations:** a Department of Clinical Laboratory, Xinchang Hospital of traditional Chinese (MD) Medicine, Shaoxing, Zhejiang Province, People’s Republic of China; b Department of Clinical Laboratory, The People’s Hospital of Cangnan Zhejiang, Wenzhou, Zhejiang Province, People’s Republic of China; c Department of Rehabilitation, Taizhou Hospital of Zhejiang Province, Taizhou, Zhejiang province, People’s Republic of China; d Department of Clinical Laboratory, Linping Campus, The Second Affiliated Hospital of Zhejiang University School of Medicine, Zhejiang Province, People’s Republic of China.

**Keywords:** C-reactive protein, influenza B, lymphocyte/monocyte ratio, neutrophil/lymphocyte ratio, platelet/lymphocyte ratio, white blood cells

## Abstract

The aim of the study was to study the diagnostic and therapeutic utility of NLR (neutrophil-to-lymphocyte ratio), LWR (lymphocyte-to-monocyte ratio), PLR (platelet-to-lymphocyte ratio), and WBC × CRP (WBC: white cell count, CRP: C-reactive protein) in patients with influenza B. This retrospective study included 122 adult patients with influenza B, 176 adult patients with bacterial infection, and 119 adult healthy physical examinees for routine blood examination and CRP testing, calculation of NLR, LMR, PLR, and WBC × CRP for relevant statistical analysis, monitoring of NLR, LMR, PLR and WBC × CRP in patients with influenza B during relevant treatment. All indicators, except for WBC and NLR, had no statistical differences between the influenza B group, the normal control group, and the influenza B group and bacterial infection group, respectively, and showed no statistical significance for the differences between the groups. The diagnostic effect of LMR and WBC × CRP was deemed good or excellent in patients with influenza B, healthy people, and patients with a bacterial infection. Conversely, NLR and PLR could only distinguish patients with influenza B from healthy people but remained unable to identify different pathogens. Moreover, many false negatives were noted for WBC and CRP during the diagnosis of influenza B. Also, NLR, LMR, PLR, and WBC × CRP exerted a good effect in evaluating curative effect and conditions for influenza B. LMR and WBC × CRP have a relatively high value in the early diagnosis of adults suffering from influenza B. Also, NLR and PLR excelled at differentiating adult patients with influenza B from healthy people. Therefore, NLR, PLR, LMR, and WBC × CRP can all be used for disease course monitoring and efficacy evaluation.

## 1. Introduction

Influenza is an acute respiratory infectious disease caused by various viruses. It has the characteristics of high infectivity, pronounced seasonality, widespread susceptibility, distinctive epidemiological history, and difficulty in management.^[1,2]^ Influenza viruses are RNA viruses of orthomyxoviridae. People are susceptible to influenza A and B viruses, which are characterized by rapid onset of symptoms. The disease is self-limited for most people; however, due to its rapid progression, some patients who fail to self-limit are susceptible to complications such as pneumonia, and the disease may progress to a severe form and result in fatalities due to acute respiratory distress syndrome, or multiple organ failure.^[3,4]^ The early administration of anti-influenza medications may substantially relieve associated symptoms. In addition, during the course of treatment, a prompt and precise evaluation of efficacy might halt the disease progression and mitigate pertinent sequelae. Therefore, early and prompt influenza diagnosis and monitoring of curative effects are critical for improving the prognosis of influenza patients.^[5]^

Typical influenza virus detection methods include virus isolation and culture, RT-PCR (reverse transcription-PCR, RT-PCR), and serological testing. However, virus isolation, culture, and serological testing are time-consuming procedures that cannot be used for outpatient screening. It was revealed that RT-PCR outperforms most other diagnostic techniques. However, this technique is expensive and has stringent equipment and environmental requirements. All these techniques are applicable to non-emergency conditions, particularly in primary hospitals. In addition, it was determined that the rapid influenza antigen test is fast, easy, and accurate; nevertheless, its sensitivity is poor and it is prone to provide a false negative result.^[6,7]^

The peripheral routine blood examination and CRP (C-reactive protein, CRP) are preferred for patients presenting fever symptoms. The routine blood examination has been listed as a laboratory test in the “Diagnosis and Treatment of Influenza (2019 edition),”^[8]^ while CRP is a classic differential diagnosis indicator of infection. During viral infection, medical textbooks also describe a decrease in WBC (white blood cell, WBC), an increase in lymphocytes, and a slight rise in CRP levels relative to baseline levels. Despite variances in the amplitude of routine blood examination changes, the majority of patients’ relevant results remain within normal reference limits. In addition, longitudinal comparisons of patients are challenging, which may impact the utilization of a single item for routine blood screening, influenza diagnosis, and efficacy evaluation. There is a slight increase in viral infection and a significant increase in bacterial infection in CRP, however it is difficult to discern whether a single small increase in CRP indicates a viral or bacterial infection(mild infection).^[9,10]^The ratio combination of the above indicators can eliminate individual variation and instability and reflect the equilibrium between the inflammatory response and immune status of the body, given that for a single indicator, as described above, there are significant variations and fluctuations among individuals, which are subject to numerous factors.

According to the majority of published studies,^[11,12]^ NLR, PLR, LMR, and other indices of the systemic inflammatory response are closely associated with the diagnosis, development, and prognosis of numerous disorders. These measures can more accurately indicate the balance between immune responses and inflammatory responses in patients. NLR has been reported most frequently in the study on the diagnosis, treatment, and prognosis of diseases. The application of blood routine indicators in children with influenza infections has been extensively investigated.^[13]^ However, relatively few studies have explored their applicability in adults. Therefore, the NLR, LWR, and PLR levels determined from normal blood tests were used to examine their relevance in the diagnosis and treatment of influenza B in adults. Therefore, this study selected adult patients infected with influenza B virus to investigate the diagnostic and therapeutic utility of CRP, routine blood examination, and related indexes (NLR, LMR, PLR, and WBC × CRP) based on their respective levels in adults infected with influenza B virus.

## 2. Materials

### 2.1. Study population

**Figure 1.**
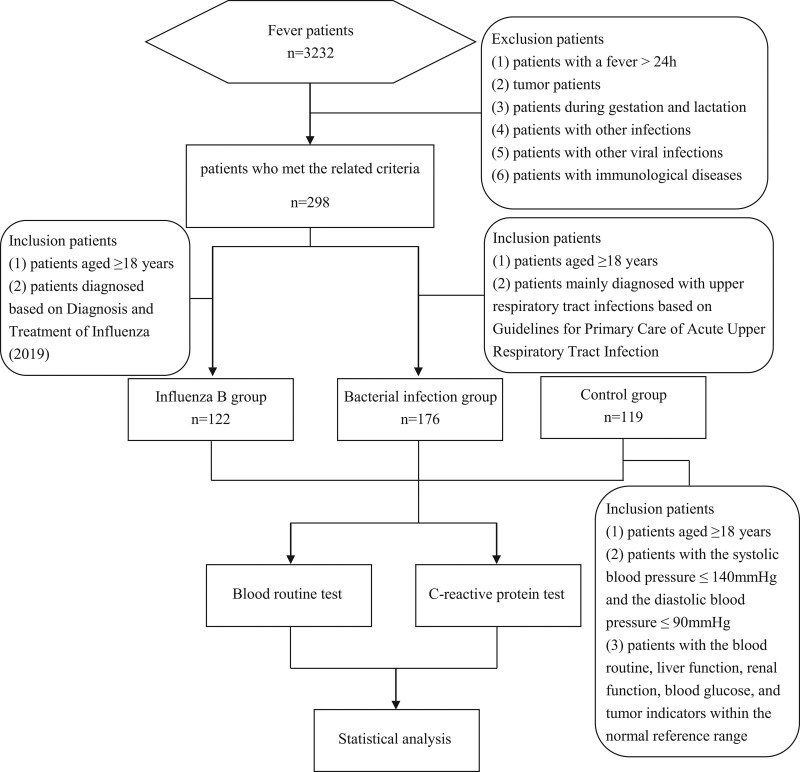
Flowchart shows the patient selection process, along with the inclusion and exclusion.

This study was approved by the institutional ethics review board. We analyzed the detection results of respiratory virus antigens (influenza A virus, influenza B virus, respiratory syncytial virus, adenovirus, and mycoplasma pneumoniae antigens) in adults with upper respiratory tract infection (body temperature > 37.3°C) who were treated at our hospital between October 2021 and January 2022. According to the statistics, the influenza B group was comprised of 122 individuals infected with the influenza B virus aged 31 (18–78) years (the greatest proportion), including 62 males and 60 females. During the same time period, the bacterial group comprised 176 instances of upper respiratory tract bacterial infection aged 32 (18–73) years, including 90 males and 86 females, while the normal control group comprised 119 healthy subjects aged 31 (18–81) years, including 61 males and 58 females. The inclusion criteria included patients with influenza B, including: patients aged ≥ 18 years and patients diagnosed based on *Diagnosis and Treatment of Influenza (2019*)^[8]^ (to determine whether the influenza B nucleic acid test is positive); patients with infectious respiratory diseases, including: patients aged ≥ 18 years and patients mainly diagnosed with upper respiratory tract infections (including acute and chronic sinusitis, acute and chronic tonsillitis, and nasopharyngitis) based on *Guidelines for Primary Care of Acute Upper Respiratory Tract Infection*^[13]^; healthy individuals, including: patients aged ≥ 18 years, patients with the systolic blood pressure ≤ 140 mmHg and the diastolic blood pressure ≤ 90 mmHg, and patients with the blood routine, liver function, renal function, blood glucose, and tumor indicators within the normal reference range. The exclusion criteria included: patients with a fever > 24 h; tumor patients; patients during gestation and lactation; patients with other infections (such as pneumonia and urinary system infections); patients with other viral infections (such as influenza A virus, respiratory syncytial virus, adenovirus, and mycoplasma pneumonia); patients with immunological diseases, hematological diseases or severe liver and kidney dysfunction (Fig. 1). On the day of admission, blood samples were drawn and preserved in ethylenediamine tetraacetic acid anticoagulant tubes, and throat swabs were also collected from these patients. These samples were sent for testing within 2 hours. In addition, the blood samples and throat swabs of patients in the influenza B group were obtained and preserved in ethylenediamine tetraacetic acid anticoagulant tubes on Days 3, 5, and 7, respectively. These samples were sent for testing within 2 hours.

### 2.2. Instruments and consumables

The influenza virus antigen (including influenza A virus antigen, influenza B virus antigen, respiratory syncytial virus antigen, adenovirus antigen, and mycoplasma pneumoniae antigen) kit (colloidal gold method) procured from the Hangzhou Genesis Biodetection & Biocontrol Ltd was used for influenza virus antigen detection. Sysmex XN 2000 completely automatic hematology analyzer (Sysmex, Japan) and related reagents were utilized for routine blood tests (white blood cells, absolute value of neutrophils, absolute value of lymphocytes, monocytes, and platelets). NLR = neutrophils/lymphocytes, LMR = lymphocytes/monocytes, PLR = platelet/lymphocyte, WBC × CRP = WBC × CRP (With CRP < 0.5 mg/L calculated as 0.5 mg/L); A PA-990Pro specialized protein analyzer was used to examine C-reactive protein (CRP) (Shenzhen Lifotronic Technology Co., Ltd.). The CRP test kit was used because it is based on immunological nephelometry, which states that soluble antigens can interact with particular antibodies to form insoluble immune complexes, which causes turbidity to occur in the reaction solution. Scattering would happen when light rays went through the reaction suspension, and a particular protein analyzer could detect it. The test sample unique protein concentration was correlated with the scattered light value, which the analyzer can determine automatically.

## 3. Methods

### 3.1. Detection of respiratory virus antigen

Strictly adhered to the procedure delineated in the instructions.

### 3.2. Routine blood examination (including white blood cells, neutrophils, lymphocytes, monocytes and platelets)

Adhere closely to the standard operating procedures for testing instruments. Before using the Sysmex XN 2000 on a daily basis, background testing and internal quality control must be conducted. Only once the equipment has been judged qualified may the samples be inspected. In the National Health Commission Clinical Laboratory Center and Clinical Laboratory Center of Zhejiang Province external quality assessment, the Sysmex XN 2000 fared remarkably well.

### 3.3. CRP test

Complete the test according to relevant SOP.

### 3.4. Treatment and outcome of patients

Patients with influenza B were given anti-influenza virus medication (oseltamivir) and dietary support. On day 7 after hospitalization, none of the patients exhibited any clinical symptoms, such as fever.

### 3.5. Statistical methods

SPSS26.0 software was used for data processing. A normality test was adopted for relevant data. The data of abnormal distribution were represented by median (M) [quartile (P25-P75)]. A Kruskal–Wallis test was used to compare the 3 groups, and a non-parametric Mann–Whitney *U* test for comparison between 2 groups. The diagnostic efficiency of WBC, CRP, NLR, LMR, PLR, and WBC × CRP was analyzed by a ROC (Receiver operating characteristic) curve. *P* < .05, denoted a statistically significant difference.

## 4. Results

### 4.1. Adult testing for the detection of 5 respiratory tract viral antigens

Positive samples for respiratory tract viral antigen were detected in 305 of 3232 patients with acute upper respiratory tract infection, with a detection rate of 9.44%. As shown in Table 1, there were 16 incidences of influenza A, influenza B, respiratory syncytial virus, adenovirus, and mycoplasma pneumoniae, with 3 coinfections.

**Table 1:** Summary on the detection of respiratory pathogens.

Ppathogen	Cases (n)	Detection rate (%)	Percentage of positive samples (%)	Total number of detected samples (n)
Influenza A virus	16	0.50	5.14	3232
Influenza B virus	248	7.67	79.74	
Respiratory syncytial virus	11	0.34	3.54	
Adenovirus	15	0.46	5.78	
Mycoplasma pneumoniae	18	0.56	5.79	
Two or more pathogens	3	0.09	0.09	

### 4.2. Differences in WBC, CRP, NLR, LWR, PLR, and WBC × CRP between different gender and age groups for all three groups

According to their median age, these patients in the influenza B group were divided into the 18 to 31-year-old group and the 32 to 78-year-old group; those in the bacterial infection group were divided into the 18 to 32-year-old group and the 33 to 73-year-old group; those in the normal control group were divided into the 18 to 31-year-old group and the 32 to 81-year-old group. Additionally, patients in the 3 groups (the influenza B group, the bacterial infection group, and the normal control group) were also divided into the male group and the female group according to different genders. The Mann–Whitney *U* test results indicated that there was no significant difference in WBC, CRP, NLR, LWR, PLR, and WBC × CRP between different gender and age groups for these 3 groups (the influenza B group, the bacterial infection group, and the normal control group) (*P* < .05) (Tables 2, 3, and 4).

**Table 2:** Comparison of WBC, CRP, NLR, LWR, PLR, and WBC × CRP between different gender and age in influenza B group.

Item	Gender				Age (yr)			
	Male	Female	Z value	*P* value	18~31	32~78	Z value	*P* value
Cases (n)	62	60	—	—	62	60	—	—
WBC (×10^9^/L)	5.5 (4.5~7.8)	6.1 (4.7~7.5)	−0.720	.472	5.6 (4.5~7.2)	5.9 (4.8~7.9)	−0.202	.840
CRP (mg/L)	7.35 (5.2~8.3)	7.55 (6.35~12.7)	−1.158	.247	7.4 (6.1~8.0)	7.6 (5.8~10.4)	−0.920	.358
NLR	4.20 (2.97~6.30)	4.60 (2.83~7.08)	−0.515	.607	3.94 (2.90~6.30)	4.77 (3.03~6.68)	−1.060	.289
LMR	1.40 (1.02~1.95)	1.52 (1.11~2.45)	−1.134	.257	1.35 (1.07~1.99)	1.62 (1.03~2.08)	−0.796	.426
PLR	175.06 (132.99~259.49)	205.80 (146.41~301.175)	−1.608	.108	199.39 (151.65~295.59)	183.26 (116.95~267.64)	−1.634	.102
WBC × CRP	37.59 (26.32~61.42)	45.15 (27.07~71.05)	−1.352	.176	40.76 (26.79 ~61.42)	42.64 (26.46~88.78)	−0.558	.577

CRP = C-reactive protein, LMR = lymphocyte-to-monocyte ratio, NLR = neutrophil-to-lymphocyte ratio, PLR = platelet-to-lymphocyte ratio, WBC = white cell count.

**Table 3:** Comparison of WBC, CRP, NLR, LWR, PLR, and WBC × CRP between different gender and age in bacterial infection group.

Item	Gender				Age (yr)			
	Male	Female	Z value	*P* value	18~32	33~73	Z value	*P* value
Cases (n)	90	86	—	—	88	88	—	—
WBC (×10^9^/L)	8.4 (6.0~11.1)	7.3 (6.1~10.1)	−0.993	.321	7.8 (6.1~10.6)	8.3 (6.0~11.1)	−0.342	.733
CRP (mg/L)	8.6 (6.9~22.1)	7.6 (6.2~21.5)	−0.896	.371	7.8 (6.2~21.5)	8.3 (6.8~22.3)	−0.527	.598
NLR	4.42 (2.60~7.22)	4.96 (2.88~6.85)	−0.370	.711	5.20 (2.94~7.19)	4.69 (2.56~6.81)	−1.604	.109
LMR	1.77 (1.26~2.34)	1.81 (1.53~2.44)	−0.857	.392	1.80 (1.32~2.41)	1.82 (1.36~2.43)	−0.111	.912
PLR	154.48 (128.57~216.85)	163.17 (130.88~224.66)	−0.530	.596	153.22 (128.43~243.53)	162.24 (132.88~221.33)	−0.238	.812
WBC × CRP	85.57 (39.96~214.12)	79.34 (38.44~206.08)	−0.798	.725	60.78 (33.75 ~210.86)	73.36 (38.71~217.04)	−0.756	.450

CRP = C-reactive protein, LMR = lymphocyte-to-monocyte ratio, NLR = neutrophil-to-lymphocyte ratio, PLR = platelet-to-lymphocyte ratio, WBC = white cell count.

**Table 4:** Comparison of WBC, CRP, NLR, LWR, PLR, and WBC × CRP between different gender and age in control group.

Item	Gender				Age (yr)			
	Male	Female	Z value	*P* value	18~31	32~81	Z value	*P* value
Cases (n)	61	58	—	—	60	59	—	—
WBC (×10^9^/L)	6.0 (5.3~7.1)	5.9 (5.1~6.8)	−0.931	.352	6.0 (5.2~7.3)	5.8 (5.2~6.8)	−1.066	.286
CRP (mg/L)	1.9 (0.8~4.6)	2.5 (1.0~6.1)	−1.387	.165	2.8 (1.0~5.3)	2.2 (0.9~5.4)	−0.266	.790
NLR	1.96 (1.52~2.83)	1.95 (1.24~2.52)	−0.904	.366	1.92 (1.37~2.39)	1.98 (1.49~2.81)	−0.460	.646
LMR	5.19 (3.99~6.80)	5.54 (3.99~7.14)	−0.816	.414	5.08 (3.80~6.56)	5.61 (4.17~7.38)	−1.377	.169
PLR	115.97 (89.35~146.49)	109.09 (82.96~137.27)	−1.435	.151	106.86 (84.54~142.60)	114.48 (88.96~143.93)	−0.712	.476
WBC × CRP	6.49 (3.00~16.93)	6.60 (2.84~16.91)	−0.332	.740	9.16 (3.00 ~19.13)	5.96 (2.87~13.98)	−1.568	.093

CRP = C-reactive protein, LMR = lymphocyte-to-monocyte ratio, NLR = neutrophil-to-lymphocyte ratio, PLR = platelet-to-lymphocyte ratio, WBC = white cell count.

### 4.3. The comparison of laboratory indicators between influenza B group, bacterial infection group, and normal control group

**Figure 2.**
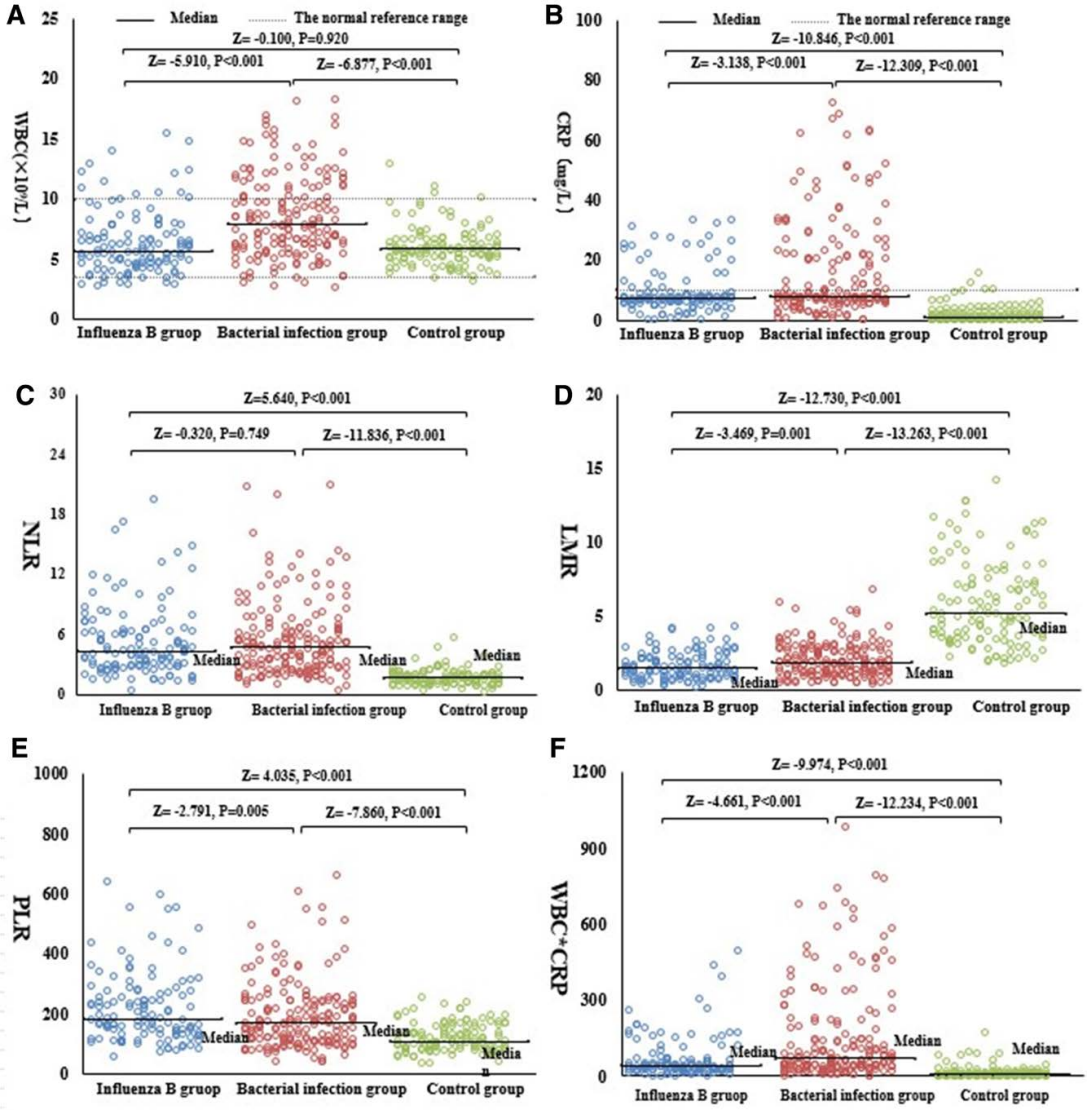
The levels of WBC, CRP, NLR, LMR, PLR, and WBC × CRP in the influenza B, bacterial infection, and control groups at admission are compared. CRP = C-reactive protein, LMR = lymphocyte-to-monocyte ratio, NLR = neutrophil-to-lymphocyte ratio, PLR = platelet-to-lymphocyte ratio, WBC = white cell count. Notes: The analyses of WBC in (A), CRP in (B), NLR in (C), LMR in (D), PLR in (E), and WBC × CRP in (F) were made with a Mann–Whitney *U* test.

Changes in WBC, CRP, NLR, LMR, PLR, and WBCCRP were statistically significant between the influenza B group, the bacterial infection group, and the normal control group (Table 5). In terms of WBC, there was no statistically significant difference between the influenza B and normal control groups. In the remaining comparisons, however, a statistically significant difference was seen between the 2 groups (Fig. 2A). For NLR, there was no statistical difference between the influenza B group and the bacterial infection group, but other comparisons between the 2 groups revealed statistical differences (Fig. 2C). There were statistically significant differences for CRP, LMR, PLR, and WBC × CRP between the influenza B group, the bacterial infection group, and the normal control group (Fig. 2B, D, E, and F).

**Table 5:** The comparison of laboratory indicators among the influenza B group, bacterial infection group and control group.

Item	Influenza B group	Bacterial infection group	Control group	H value	*P* value
Cases (n)	122	176	119	—	—
WBC (×10^9^/L)	5.6 (4.7~7.8)	7.9 (6.1~11.0)	5.8 (5.1~6.7)	58.227	<.001
CRP (mg/L)	7.5 (5.9~9.4)	7.9 (6.6~22.0)	1.1 (0.5~3.0)	180.602	<.001
NLR	4.30 (2.92~6.49)	4.75 (2.82~6.70)	1.68 (1.22~2.07)	169.412	<.001
LMR	1.45 (1.04~2.05)	1.80 (1.34~2.42)	5.27 (3.99~6.90)	225.273	<.001
PLR	186.23 (141.47~273.51)	158.14 (129.12~222.88)	112.22 (86.14~144.07)	89.784	<.001
WBC × CRP	41.00 (26.53~69.08)	70.62 (38.84~213.09)	6.60 (2.95~16.64)	176.937	<.001

WBC, CRP, NLR, LMR, PLR, and WBC × CRP adopted Kruskal–Wallis H-test.

CRP = C-reactive protein, LMR = lymphocyte-to-monocyte ratio, NLR = neutrophil-to-lymphocyte ratio, PLR = platelet-to-lymphocyte ratio, WBC = white cell count.

### 4.4. The value of NLR, LMR, PLR, and WBC × CRP for differential diagnosis in patients with influenza B and healthy people

**Figure 3.**
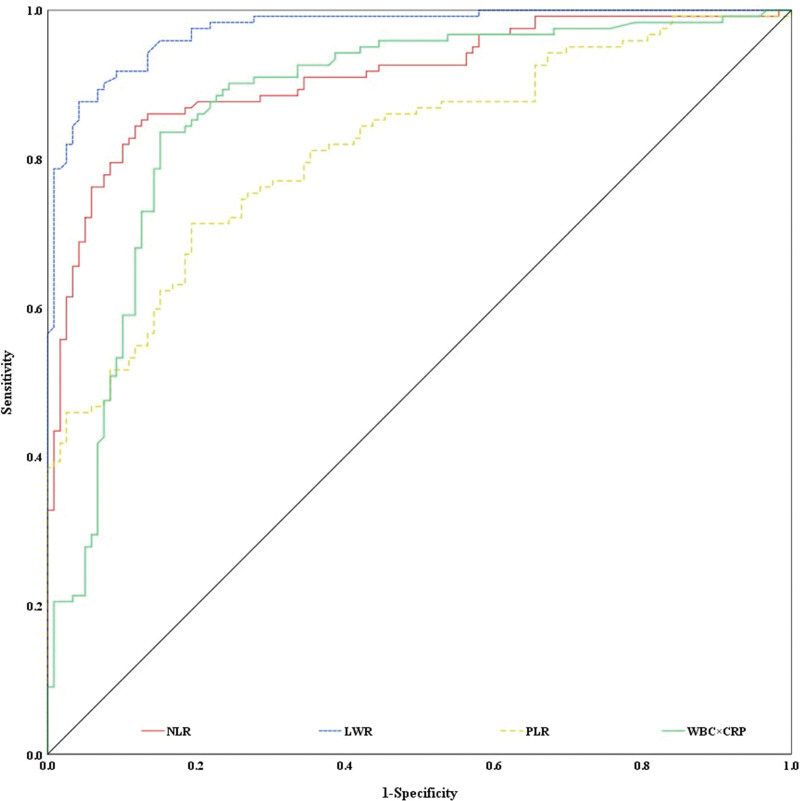
ROC curves of NLR, LMR, PLR, and WBC × CRP for the differential diagnosis in patients with influenza B and healthy people. CRP = C-reactive protein, LMR = lymphocyte-to-monocyte ratio, NLR = neutrophil-to-lymphocyte ratio, PLR = platelet-to-lymphocyte ratio, ROC = receiver operating characteristic, WBC = white cell count.

The ROC curves of NLR, LMR, PLR and WBC × CRP showed that the AUCs (area under the curve, 95% confidence interval) of NLR, LMR, PLR, and WBC × CRP for differential diagnosis in patients with influenza B and healthy people were 0.910 (0.873~0.948), 0.974 (0.985~0.991), 0.813 (0.760~0.867) and 0.872 (0.824~0.919) respectively. The optimal critical values were 2.513, 2.675, 147.993, and 22.375, respectively (Fig. 3). NLR, LMR, PLR, and WBC × CRP had relatively high diagnostic power, while LMR had the highest diagnostic power. WBC and CRP had a low diagnostic power (Table 6).

**Table 6:** Diagnostic power of WBC, CRP, NLR, LMR, PLR, and WBC × CRP in patients with influenza B and healthy people.

Item	Cut off value	Sensitivity (%)	Specificity (%)	Positive predictive value (%)	Negative predictive value (%)	Youden index	Accuracy (%)
WBC (×10^9^/L)	4~10	25.41	91.60	75.61	54.50	17.01	58.09
CRP (mg/L)	<10	22.13	96.64	87.10	54.76	18.77	58.92
NLR	2.513	86.89	87.39	87.60	86.67	74.28	87.14
LMR	2.675	87.70	95.80	95.54	88.37	83.50	91.70
PLR	147.993	71.31	80.67	79.09	73.28	51.98	75.93
WBC × CRP	22.375	83.61	84.87	85.00	84.87	68.48	84.23

CRP = C-reactive protein, LMR = lymphocyte-to-monocyte ratio, NLR = neutrophil-to-lymphocyte ratio, PLR = platelet-to-lymphocyte ratio, WBC = white cell count.

### 4.5. The value of WBC, CRP, NLR, LMR, PLR, and WBC × CRP for differential diagnosis in patients with influenza B and bacterial infection

**Figure 4.**
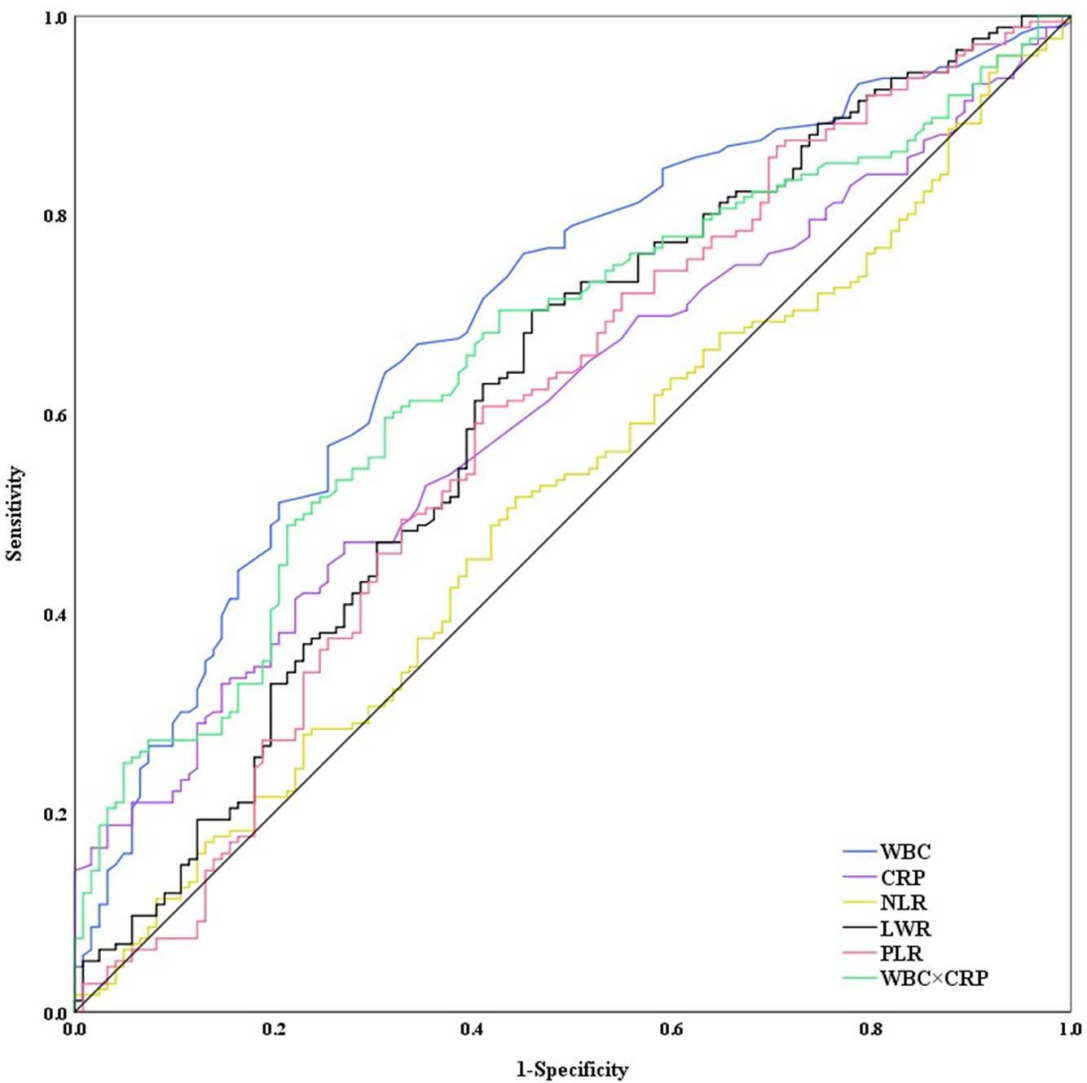
ROC curves of WBC, CRP, NLR, LMR, PLR, and WBC × CRP for differential diagnosis in patients with influenza B and bacterial infection. CRP = C-reactive protein, LMR = lymphocyte-to-monocyte ratio, NLR = neutrophil-to-lymphocyte ratio, PLR = platelet-to-lymphocyte ratio, ROC = receiver operating characteristic, WBC = white cell count.

The ROC curves of WBC, CRP, NLR, LMR, PLR and WBC × CRP showed that the AUCs (95% confidence interval) of WBC, CRP, NLR, LMR, PLR and WBC × CRP for differential diagnosis in patients with patients with influenza B and healthy people were 0.701 (0.641~0.761), 0.607 (0.543~0.670), 0.511 (0.444~0.571), 0.618 (0.552~0.684), 0.595 (0.528~0.662) and 0.659 (0.597~0.721) respectively, The optimal critical values were 6.75, 8.5, 4.625, 1.524, 174.299 and 53.065 respectively (Fig. 4). WBC, CRP, LMR, and WBC × CRP possessed a good capacity for diagnosis. In contrast, NLR and PLR have poor diagnostic accuracy (Table 7).

**Table 7:** Value of WBC, CRP, NLR, LWR, PLT and WBC × CRP for differential diagnosis in patients with influenza B and bacterial infection.

Item	Cut off value	Sensitivity (%)	Specificity (%)	Positive predictive value (%)	Negative predictive value (%)	Youden index	Accuracy (%)
WBC (×10^9^/L)	6.75	64.20	68.85	74.83	57.14	33.06	66.11
CRP (mg/L)	8.5	53.41	72.95	74.02	52.05	26.36	61.41
NLR	4.625	57.95	55.74	65.38	47.89	13.69	57.05
LMR	1.524	70.45	54.10	68.89	55.93	24.55	63.76
PLR	174.299	60.80	40.16	59.44	41.53	0.96	52.35
WBC × CRP	53.065	59.66	68.85	73.43	68.85	28.51	63.42

CRP = C-reactive protein, LMR = lymphocyte-to-monocyte ratio, NLR = neutrophil-to-lymphocyte ratio, PLR = platelet-to-lymphocyte ratio, WBC = white cell count.

### 4.6. Dynamic changes of NLR, LMR, PLR, and WBC × CRP during diagnosis and treatment in the influenza B group

**Figure 5.**
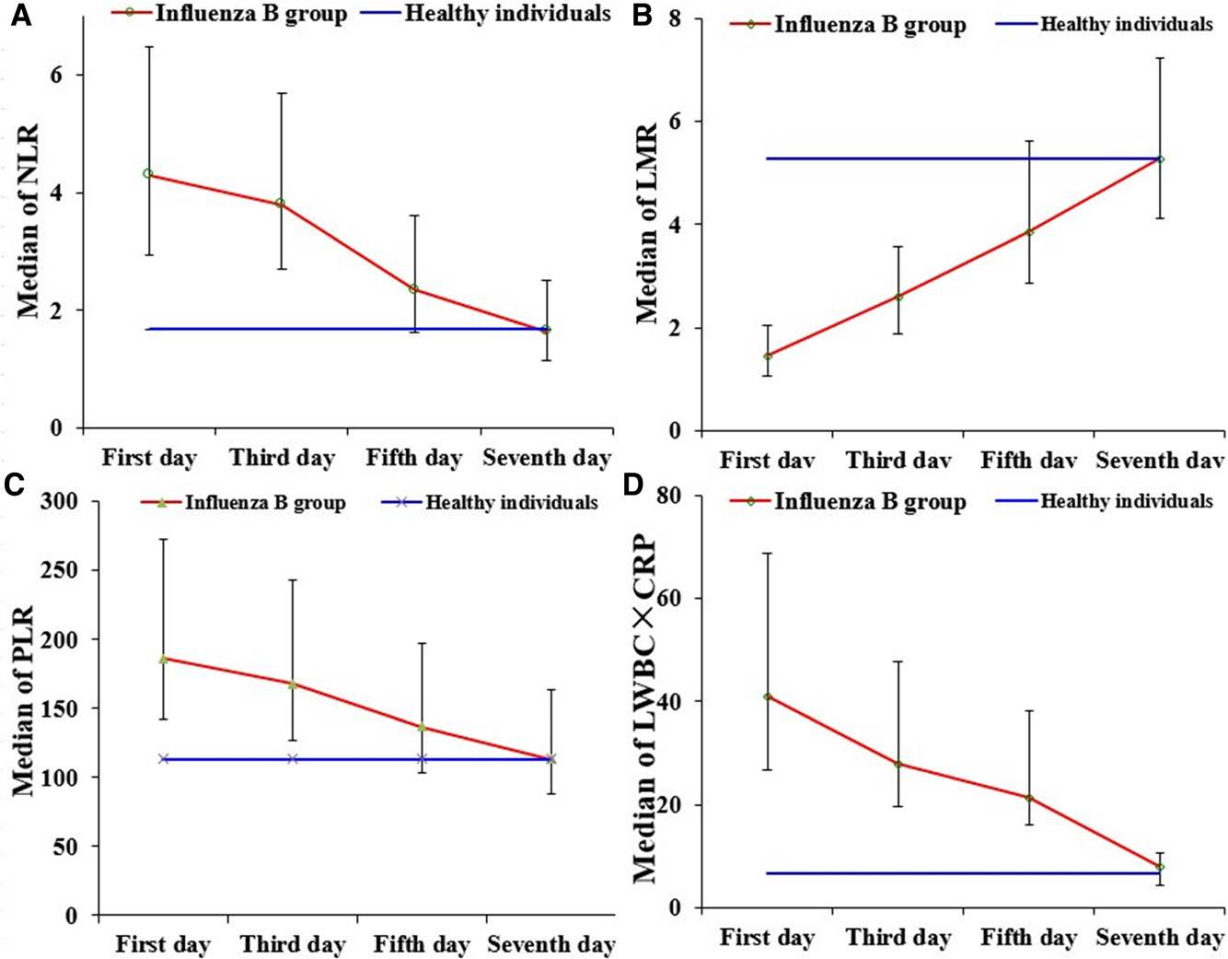
The dynamic changes of NLR, LMR, PLR, and WBC × CRP in the influenza B group. CRP = C-reactive protein, LMR = lymphocyte-to-monocyte ratio, NLR = neutrophil-to-lymphocyte ratio, PLR = platelet-to-lymphocyte ratio, WBC = white cell count, Notes: a: NLR, b: LMR, c: PLR, d: WBC × CRP.

Effective treatment (complete evaluation) considerably improved NLR, LMR, PLR, and WBC × CRP until Day 5 and Day 7, when they recovered to near-normal and normal levels, respectively (Fig. 5).

## 5. Discussion

Several sensitive markers presently utilized in clinical practice for routine blood examination and CRP testing can be applied to clinical diagnosis, differential diagnosis, and effectiveness evaluation.^[14,15]^ A bacterial infection may result in an increase in the ratio of white blood cells to neutrophils as well as a large increase in CRP. According to textbooks, viral infections may cause a decrease in the ratio of white blood cells to neutrophils and a slight increase in C-reactive protein. The aforementioned changes may only be detected by comparing the onset conditions to the present health status of the patient. However, it is impossible to do frequent routine blood and CRP testing on the vast majority of individuals. Therefore, current standard blood examination and CRP testing results cannot be provided, making it difficult to compare pre- and post-intervention symptoms. For disease diagnosis and differential diagnosis, clinically, test results are compared to the normal reference interval. Due to the vast reference ranges of routine blood indicators and CRP in some patients, misinterpretation may occur if baseline values are within the middle-lower reference ranges and rise to the upper limit of the reference ranges after pathogen infection. Reestablishing reference intervals for various indications and establishing additional dependable diagnostic indicators are the main options for the aforementioned challenges. Routine blood examination and CRP testing have been utilized in the diagnosis or differential diagnosis of a variety of disorders, with widely established reference intervals. This is because they are the most often used traditional measures in clinical practice. Establishing a reference interval for a specific disease in a specific population is therefore unscientific and impractical. The combination of regular blood markers and CRP is extremely important for diagnosing and treating influenza B patients.

In this study, the normal reference intervals for WBC (4~10 × 10)^9^/L) and CRP (<10mg/L) were cutoff values of low sensitivity in the differential diagnosis of patients with influenza B and healthy people. The rate of incorrect diagnosis by a single WBC or CRP will approach 50%. In contrast to the findings of this investigation, Yi Liao^[16]^ et al found that WBC had a high diagnostic power in children with influenza A, and Shui Fu^[17]^ et al determined that CRP likewise had a high diagnostic power in children with influenza A. They had all utilized the ROC curve to determine the respective cutoff values. Inconsistent cutoff values led to large diagnostic power disparities. Normal reference intervals are frequently employed in clinical settings as the cutoff values adopted for this investigation. The ROC curve based on the reference range of WBC (4~10 × 10^9^/L) has a wider cutoff value because WBC is an established and often used experimental index. The accuracy of WBC in differentially diagnosing patients with influenza B would be impacted by an increase in false positives and negatives as a result. Based on the ROC curves, this study found that WBC and CRP had a high diagnostic power in influenza B and bacterial infection; however, the corresponding cutoff values were still within the normal reference range (WBC: 6.75 × 10^9^/L, CRP: 8.5mg/L). Therefore, their clinical use for distinguishing influenza B virus infection from bacterial infection is negligible. Based on this conclusion, the value of WBC and CRP for differential diagnosis in patients with influenza B is low, which may even lead to high misdiagnosis rates. Therefore, this study carried out a ratio combination of CRP and routine blood indicators to explore more valuable indicators for diagnosing and treating patients suffering from influenza B.

In recent years, a number of studies have demonstrated the utility of NLR, LMR, and PLR as novel predictive markers for the early detection and diagnosis of infectious disorders, such as viral infections. Nevertheless, few studies have investigated adult influenza virus infections.^[14,16,18]^ In addition, no evidence supports the use of WBC × CRP in the diagnosis and treatment of infectious diseases. Theoretically, bacterial infections can increase WBC and significantly decrease CRP, but viral infections can cause a decrease in WBC and a slight increase in CRP. Therefore, it can be assumed that WBC × CRP would only slightly increase in patients with viral infections (due to the small range of stable WBC and the wide range of CRP with a lower normal value contributing to a smaller decrease of WBC compared to the increase of CRP in clinical practice) and significantly increase in patients with bacterial infections (both WBC and CRP increase, and CRP increases significantly). Pathogen infections are the primary cause of fever in most individuals. Therefore, the purpose of this study is to investigate the role of WBC × CRP in the differential diagnosis and management of influenza B patients. This study shown that NLR, LMR, PLR, and WBC × CRP have good diagnostic value for distinguishing influenza B from healthy individuals. Therefore, these numbers could be utilized to differentiate between influenza B victims and healthy individuals. This conclusion is generally compatible with the research of Ronghe Zhu^[14]^ and Yi Liao.^[16]^ In addition, this study investigated the differential diagnostic effectiveness of NLR, LMR, PLR, and WBC × CRP in the differential diagnosis of influenza B and bacterial infection and discovered that both LMR and WBC had a high diagnostic capacity for differential diagnosis. In contrast, NLR and PLR showed low diagnostic power as a result of their weak pathogen recognition. The majority of studies on the diagnostic potential of NLR, LMR, and PLR are limited to patients infected with influenza virus and healthy individuals; thus, the markers are infrequently applied to the differential diagnosis of various diseases. Likewise, there is still no report about WBC × CRP. Therefore, it is impossible to compare this study to earlier publications in this regard. Nonetheless, this research can fill the need in this sector. An exhaustive examination of the diagnostic capability of NLR, LMR, PLR, and WBC × CRP in physiological and pathological situations based on different pathogens revealed that NLR and PLR can differentiate between physiological and pathological conditions but cannot differentiate between distinct pathogens. Those findings are consistent with the results of YUAN Xiao-hong et al^[19]^ However, LMR and WBC × CRP can distinguish between healthy and pathological conditions and differentiate between bacterial and viral infections. LMR and WBC × CRP are therefore of greater clinical importance than NLR and PLR. Consequently, they are the first option for differential diagnosis of influenza B virus infection.

The dynamic monitoring of changes in NLR, LMR, PLR, and WBC × CRP among patients with influenza B during diagnosis and treatment has found that effective treatment can alleviate relevant clinical symptoms not only rapidly but also quickly restore NLR, LMR, PLR, and WBC × CRP to normal levels. This study has suggested that it takes about 5 days for NLR, LMR, PLR, and WBC × CRP to return to approximately normal levels and 7 days to normal levels, which is consistent with the pathophysiological process of the influenza B virus. Moreover, the study has further proved that NLR, LMR, PLR, and WBC × CRP play an important role in the monitoring of influenza B, and NLR, LMR, PLR, and WBC × CRP can be used as indicators for the evaluation of curative effects as well as whole-process monitoring for patients suffering from influenza B. When the indicators have been altered, there should be a comprehensive evaluation of relevant changes in organisms and treatment options for preventing complications, thereby avoiding disease exacerbation. Usually, the first diagnosis for patients with influenza B is made in grass-roots hospitals for emergency treatment. However, it is difficult for grassroots hospitals to carry out more infection-related testing due to restricted resources, leading to a conflict between detection level and clinical demand. Local NLR, LMR, PLR, and WBC × CRP reference intervals should be established wherever possible for the grass-roots hospitals. Moreover, clinicians should first conduct a preliminary determination on LMR and WBC × CRP efficiently. Moreover, there should be a comprehensive diagnosis in combination with NLR, PLR, WBC, and CRP. The patients diagnosed with influenza B should be continuously monitored for relevant changes during treatment and evaluated for outcomes and conditions.

## 6. Conclusion

The results of this study show that LMR and WBC × CRP are highly useful diagnostic markers for the identification and management of adult patients with influenza B. However, this investigation was conducted using a small sample size, and no comparisons to other viral infections were made. Therefore, additional research is required to confirm the clinical utility of LMR and WBC × CRP in the diagnosis and care of adult patients with influenza B. Regarding WBC and CRP, the considerable risk of misdiagnosis due to false-negative readings should not be neglected. NLR and PLR can differentiate between pathological and physiological circumstances and can therefore be utilized to assess the outcomes and conditions of influenza B adult patients. As NLR, LMR, PLR, and WBC × CRP are all adopted, routine blood examination and CRP testing can be performed even in community hospitals. Consequently, a comprehensive analysis on WBC, CRP, NLR, LMR, PLR, and WBC × CRP can be adopted for complementary advantages, to guarantee the diagnosis and treatment of influenza B adult patients.

## Acknowledgments

We would like to acknowledge the hard and dedicated work of all the staff that implemented the intervention and evaluation components of the study.

## Author contributions

**Conceptualization:** Juan-Fei Qi, Shui Fu, Liu-Ling Chen.

**Formal analysis:** Juan-Fei Qi.

**Funding acquisition:** Mei-Li Guo.

**Investigation:** Liu-Ling Chen.

**Software:** Juan-Fei Qi, Li Lin.

**Supervision:** Liu-Ling Chen.

**Writing – original draft:** Juan-Fei Qi.

**Writing – review & editing:** Juan-Fei Qi, Shui Fu, Liu-Ling Chen.

## References

[1] Ryu S Cowling BJ. Human Influenza Epidemiology. Cold Spring Harb Perspect Med. 2021;11:a038356. 32988982 10.1101/cshperspect.a038356 PMC8634793

[2] Krammer F Smith GJD Fouchier RAM. Influenza. Nat Rev Dis Primers. 2018;4:3. 29955068 10.1038/s41572-018-0002-y PMC7097467

[3] Frenzen F. Der Mensch und Influenza – einÜberblick [Human and Influenza - an Overview]. Pneumologie. 2018;72:207–21. German. 29514356 10.1055/s-0043-105850

[4] Yang J Lau YC Wu P. Variation in Influenza B Virus epidemiology by lineage, China. Emerg Infect Dis. 2018;24:1536–40. 30015611 10.3201/eid2408.180063 PMC6056115

[5] Gaitonde DY Moore FC Morgan MK. Influenza: diagnosis and treatment. Am Fam Physician. 2019;100:751–8. 31845781

[6] Merckx J Wali R Schiller I. Diagnostic accuracy of novel and traditional rapid tests for influenza infection compared with reverse transcriptase polymerase chain reaction: a systematic review and meta-analysis. Ann Intern Med. 2017;167:394–409. 28869986 10.7326/M17-0848

[7] Bruning AHL Leeflang MMG Vos JMBW. Rapid tests for influenza, respiratory syncytial virus, and other respiratory viruses: a systematic review and meta-analysis. Clin Infect Dis. 2017;65:1026–32. 28520858 10.1093/cid/cix461 PMC7108103

[8] National Health Commission of the People’s Republic of China; State Administration of Traditional Chinese Medicine. Protocol for diagnosis and treatment of influenza (2019 version). Chin J Clin Infect Dis. 2019;12:451–5.

[9] Bernstein D Coster D Berliner S. C-reactive protein velocity discriminates between acute viral and bacterial infections in patients who present with relatively low CRP concentrations. BMC Infect Dis. 2021;21:1210. 34863104 10.1186/s12879-021-06878-y PMC8643010

[10] Li Y Min L Zhang X. Usefulness of procalcitonin (PCT), C-reactive protein (CRP), and white blood cell (WBC) levels in the differential diagnosis of acute bacterial, viral, and mycoplasmal respiratory tract infections in children. BMC Pulm Med. 2021;21:386. 34836530 10.1186/s12890-021-01756-4 PMC8620633

[11] Saumet L Deschamps F Marec-Berard P. Radiofrequency ablation of metastases from osteosarcoma in patients under 25 years: the SCFE experience. Pediatr Hematol Oncol. 2015;32:41–9. 25007012 10.3109/08880018.2014.926469

[12] Yevich S Gaspar N Tselikas L. Percutaneous computed tomography-guided thermal ablation of pulmonary osteosarcoma metastases in children. Ann Surg Oncol. 2016;23:1380–6. 26589502 10.1245/s10434-015-4988-z

[13] Chinse Medical Association, Chinese Medical Journals Publishing House, Chinese Society of General Practice, Infection Group of Chinese Thoracic Society, Editorial Board of Chinese Journal of General Practitioners of Chinese Medical Association, Expert Group of Guidelines for Primary Care of Respiratory System Disease. Guideline for primary care of acute upper respiratory tract infection (2018). Chin J Gen Pract. 2019;18:422–426.

[14] Zhu R Chen C Wang Q. Routine blood parameters are helpful for early identification of influenza infection in children. BMC Infect Dis. 2020;20:864. 33213395 10.1186/s12879-020-05584-5 PMC7676412

[15] Vasileva D Badawi A. C-reactive protein as a biomarker of severe H1N1 influenza. Inflamm Res. 2019;68:39–46. 30288556 10.1007/s00011-018-1188-x PMC6314979

[16] Liao Y Liu C He W. Study on the Value of Blood Biomarkers NLR and PLR in the clinical diagnosis of influenza a virus infection in children. Clin Lab. 2021;67:2540–7. 10.7754/Clin.Lab.2021.210319 34758224

[17] Fu S Zhang MM Zhang L. The value of combined serum amyloid A Protein and Neutrophil-to-Lymphocyte Ratio Testing in the Diagnosis and Treatment of Influenza A in Children. Int J Gen Med. 2021;14:3729–35. 34326659 10.2147/IJGM.S313895 PMC8314685

[18] Ling Z Liyuan S Wei L. The diagnostic efficacy of LWR, NLR, LMR and PLR in severe cases of coronavirus disease2019. Int J Virol. 2020;27:187–90.

[19] Xiao-hong Y Peng L. Application of neutrophils to lymphocytes ratio for influenza diagnosis of children aged not more than six years old. CJCHC. 2019;27:208–11.

